# Sex-Specific Associations between Prenatal Exposure to Bisphenols and Phthalates and Infant Epigenetic Age Acceleration

**DOI:** 10.3390/epigenomes8030031

**Published:** 2024-08-10

**Authors:** Gillian England-Mason, Sarah M. Merrill, Jiaying Liu, Jonathan W. Martin, Amy M. MacDonald, David W. Kinniburgh, Nicole Gladish, Julia L. MacIsaac, Gerald F. Giesbrecht, Nicole Letourneau, Michael S. Kobor, Deborah Dewey

**Affiliations:** 1 Department of Pediatrics, Cumming School of Medicine, University of Calgary, Calgary, AB T2N 1N4, Canada; 2 Owerko Centre, Alberta Children’s Hospital Research Institute, University of Calgary, Calgary, AB T2N 1N4, Canada; 3 Department of Psychiatry and Human Behavior, The Warren Alpert Medical School at Brown University, Providence, RI 02903, USA; 4 Department of Medical Genetics, British Columbia Children’s Hospital Research Institute, University of British Columbia, Vancouver, BC V6T 1Z4, Canada; 5 Centre for Molecular Medicine and Therapeutics, Vancouver, BC V6H 0B3, Canada; 6 Department of Laboratory Medicine and Pathology, University of Alberta, Edmonton, AB T6G 2R3, Canada; 7 Science for Life Laboratory, Department of Environmental Science, Stockholm University, 114 19 Stockholm, Sweden; 8 Alberta Centre for Toxicology, University of Calgary, Calgary, AB T2N 1N4, Canada; 9 Department of Psychology, Faculty of Arts, University of Calgary, Calgary, AB T2N 1N4, Canada; 10 Department of Community Health Sciences, Cumming School of Medicine, University of Calgary, Calgary, AB T2N 1N4, Canada; 11 Faculty of Nursing, University of Calgary, Calgary, AB T2N 1N4, Canada; 12 Department of Psychiatry, Cumming School of Medicine, University of Calgary, Calgary, AB T2N 1N4, Canada; 13 Hotchkiss Brain Institute, Calgary, AB T2N 4N1, Canada; 14 Program in Child and Brain Development, Canadian Institute for Advanced Research (CIFAR), Toronto, ON M5G 1M1, Canada

**Keywords:** bisphenol A, phthalates, DNA methylation, epigenetic clock, developmental origins of health and disease, APrON

## Abstract

We examined whether prenatal exposure to two classes of endocrine-disrupting chemicals (EDCs) was associated with infant epigenetic age acceleration (EAA), a DNA methylation biomarker of aging. Participants included 224 maternal–infant pairs from a Canadian pregnancy cohort study. Two bisphenols and 12 phthalate metabolites were measured in maternal second trimester urines. Buccal epithelial cell cheek swabs were collected from 3 month old infants and DNA methylation was profiled using the Infinium MethylationEPIC BeadChip. The Pediatric-Buccal-Epigenetic tool was used to estimate EAA. Sex-stratified robust regressions examined individual chemical associations with EAA, and Bayesian kernel machine regression (BKMR) examined chemical mixture effects. Adjusted robust models showed that in female infants, prenatal exposure to total bisphenol A (BPA) was positively associated with EAA (*B* = 0.72, 95% CI: 0.21, 1.24), and multiple phthalate metabolites were inversely associated with EAA (Bs from −0.36 to −0.66, 95% CIs from −1.28 to −0.02). BKMR showed that prenatal BPA was the most important chemical in the mixture and was positively associated with EAA in both sexes. No overall chemical mixture effects or male-specific associations were noted. These findings indicate that prenatal EDC exposures are associated with sex-specific deviations in biological aging, which may have lasting implications for child health and development.

## 1. Introduction

Bisphenols and phthalates are environmental endocrine disruptors widely used in the manufacture of plastic products. Bisphenols, such as bisphenol A (BPA), are used to fabricate polycarbonate plastic products (e.g., single use plastics, baby bottles) and epoxy resins [1]. Phthalates comprise a large group of chemicals that are used to increase the malleability of plastics, and are found in self-care products, construction materials, textiles, electronics, and toys [2]. Bisphenols and phthalates are two classes of endocrine-disrupting chemicals (EDCs). They exhibit non-persistence when they are absorbed orally as they have short biological half-lives and are quickly metabolized and eliminated from the body [3,4]. The prevalence of these chemicals in everyday items results in widespread exposure in humans, including among pregnant women in Canada [5,6].

The adverse effects of gestational exposure to bisphenols and phthalates on the health of pregnant women and developing children is being increasingly documented. For example, maternal urinary concentrations of BPA and phthalate metabolites have been associated with increased risk of pregnancy complications (e.g., preeclampsia) [7,8]. Bisphenols and phthalates can cross the placenta [9,10], and in utero exposure has been associated with adverse birth outcomes, such as increased risk of preterm birth, and altered birthweight [11,12]. Further, there is evidence that maternal urinary concentrations of these chemicals during pregnancy are linked to sex-specific changes in offspring growth and development [13]. For instance, exposure to bisphenols and phthalates during pregnancy has been associated with sexually dimorphic markers (e.g., anogenital distance) in infants [14] and sex-specific neurodevelopmental outcomes in young children [15,16,17].

To illuminate the molecular processes that may be associated with risk and resilience to environmental insults, epidemiological studies are needed that identify epigenetic markers associated with early environmental chemical exposures [18]. Recent epigenome-wide association studies have shown that prenatal exposure to BPA and phthalates is associated with DNA methylation at individual methylation sites (i.e., CpGs) [19,20,21,22,23]. Further, there is some evidence indicating that prenatal exposure to BPA and phthalates such as di(2-ethylhexyl) phthalate (DEHP) is associated with sex-specific epigenomic responses [24,25], and some of the identified CpGs had been previously found to predict DNA methylation-based age in children and adolescents [23]. However, there has been little investigation as to whether prenatal exposure to EDCs is related to epigenetic aging biomarkers based on DNA methylation patterns. Improved understanding of age-related epigenetic processes that are impacted by environmental chemical exposures may aid in the development of future prevention and intervention strategies.

Epigenetic clocks estimate biological epigenetic age based on DNA methylation levels at a set of computationally derived age-related CpGs, and may be useful for identifying molecular targets for early interventions [26]. The common estimate, epigenetic age acceleration (EAA), is the residual extracted from a linear regression of DNA methylation-based age on chronological age and thus can be used to determine if the tissue is aging ‘faster’ or ‘slower’ than expected. It has been suggested that deviations in tissue-based age are likely associated with developmental trajectories, developmental disorders (e.g., autism spectrum disorder), and environmental factors that may speed up or delay biological aging [27]. One recent study examined maternal urinary concentrations of phthalates and epigenetic age in mother–child pairs from the Center for the Health Assessment of Mothers and Children of Salinas (CHAMACOS) cohort. They found that prenatal DEHP was associated with decreased intrinsic EAA, estimated using the Horvath pan-tissue clock, among males at 7 years of age [28]. A marginal negative association was also reported between the phthalate mixture and another aging biomarker, DNA methylation-based gestational age acceleration estimated using Bohlin’s clock, among males at birth [28]. However, the impact of prenatal exposure to bisphenols on EAA is currently unknown, and additional studies are needed to characterize the associations between prenatal exposure to EDCs and EAA both in pediatric populations and with tools appropriate for samples from early development.

There are complex age-related changes in DNA methylation, and most epigenetic clocks were developed and have been used with adult populations [29,30,31,32]. The Pediatric-Buccal-Epigenetic (PedBE) clock was specifically designed to measure buccal epithelial cell DNA methylation-based age and is an appropriate estimator of biological age in pediatric samples [33]. Pediatric-specific epigenetic clocks, such as PedBE, typically outperform clocks developed using adult samples (i.e., Horvath pan-tissue) at estimating children’s biological ages [27]. To our knowledge, no prior investigation has examined the associations between prenatal exposure to bisphenols and phthalates and PedBE EAA. To address this gap in knowledge, we utilized data from 224 maternal–infant pairs enrolled in the Alberta Pregnancy Outcomes and Nutrition (APrON) study to examine the associations between concentrations of 14 EDCs, including two bisphenols and 12 phthalate metabolites, and PedBE EAA in 3-month-old infants. We hypothesized that prenatal exposure to these chemicals would be associated with PedBE EAA in infants, with patterns specific to infant sex.

## 2. Results

### 2.1. Description of Sample Population

Characteristics of the maternal–child pairs are presented for the entire study sample and sub-groups, stratified by infant sex (Table 1). The mothers were primarily White (84.4%), well-educated (72.8% had a university degree), and of middle class or above social identity (81.7% had an annual family income of ≥70,000 Canadian dollars) (Table 1). Women were on average 32.0 years old (SD: ±4.3) and had an average pre-pregnancy BMI of 24.7 kg/m^2^ (SD: ±4.9). Women were mainly multiparous (57.6%) and 54.0% had male infants. For infants, the average gestational age at birth was 39.0 weeks (SD: ±1.8) and the average birthweight was 3368 g (SD: ±541) (Table 1). Analysis of variance (ANOVA) and chi-squared (χ^2^) tests revealed one significant difference between the sex-stratified groups; more women with male infants were primiparous (66.1%) than women with female infants (47.6%) (χ^2^ (1, N = 224) = 7.00, *p* = 0.01) (Table 1).

### 2.2. Bisphenol and Phthalate Metabolite Concentrations

Descriptive statistics for maternal urinary bisphenol and phthalate metabolite concentrations are reported for mothers of female infants (n = 103) (Table 2) and male infants (n = 121) (Table 3). The geometric means (GMs) of total BPA and total BPS were 1.59 and 0.179 μg/L for mothers of female infants, and 1.21 and 0.155 μg/L for mothers of male infants, respectively. The proportions of mothers of female infants who had urinary BPA and BPS concentrations above the LOD (0.32 μg/L for BPA and 0.10 μg/L for BPS) were 96.1% and 61.2%, respectively; for mothers of male infants, they were 91.8% and 53.7%, respectively. For the phthalate metabolites, mono-ethyl phthalate (MEP) had the highest GM (48.4 μg/L for mothers of female infants and 68.3 μg/L for mothers of male infants), whereas mono-carboxy-isononyl phthalate (MCNP) had the lowest GM (1.04 μg/L for mothers of female infants and 0.923 μg/L for mothers of male infants). For mothers of female infants, the molar sum of high molecular weight phthalates (ΣHMWPs), low molecular weight phthalates (ΣLMWPs), and di(2-ethylhexyl) phthalate (ΣDEHP) had GMs of 0.109, 0.471, and 0.0909 μmol/L, respectively. For mothers of male infants, the ΣHMWPs, ΣLMWPs, and ΣDEHP had GMs of 0.160, 0.615, and 0.0733 μmol/L, respectively. The proportion of women who had urinary phthalate metabolite concentrations above the LOD (0.10 μg/L) was 88.4% or greater for each metabolite analyzed. Many of the average bisphenol and phthalate metabolite concentrations did not significantly differ between the sex-stratified groups, except for some of the DEHP metabolites and the ΣHMWPs and ΣDEHP. For these metabolites and molar sums, higher average concentrations were found in mothers of female infants (e.g., GMs of MEHHP were 12.1 and 9.88 in mothers of female and male infants, respectively, GMs of ΣDEHP were 0.0909 and 0.0733 in mothers of female and male infants, respectively) (for more detail see Table S1).

### 2.3. Epigenetic Age Acceleration (EAA)

Descriptive analysis showed that EAA was approximately normally distributed and centered around zero in the overall sample (Figure S1). The distribution of EAA was similar for female and male infants (Figure S2).

### 2.4. Associations between EDCs and EAA

Sex-specific associations were found between log2-transformed EDC concentrations and EAA (Table 4). In female infants, higher prenatal exposure to BPA was associated with increased EAA (*B* = 0.72, 95% CI: 0.21, 1.24, *p* = 0.01, adjusted *p* = 0.04; i.e., for each doubling in BPA, there was a 0.72 week increase in biological age). Multiple phthalate metabolites were inversely associated with EAA in female infants. Specifically, higher prenatal exposure to MMP (*B* = −0.66, 95% CI: −1.28, −0.04, *p* = 0.04, adjusted *p* = 0.06), MCOP (*B* = −0.55, 95% CI: −1.01, −0.08, *p* = 0.02, adjusted *p* = 0.05), and MNP (*B* = −0.36, 95% CI: −0.69, −0.02, *p* = 0.04, adjusted *p* = 0.07) were associated with decreased EAA in female infants (i.e., for each doubling in these metabolites, there was a 0.36 to 0.66 week decrease in biological age). All associations survived false discovery rate (FDR) correction (adjusted *p* < 0.10).

In male infants, there was a marginal association (i.e., unadjusted *p* < 0.10) between prenatal BPA and EAA, suggesting that higher prenatal exposure to BPA may be associated with increased EAA (*B* = 0.52, 95% CI: −0.06, 1.10, *p* = 0.08, adjusted *p* = 0.54). No other associations were noted.

### 2.5. Mixture Analyses

Bayesian kernel machine regression (BKMR) analyses examined the overall EDC mixture effect on EAA in female and male infants. For female infants, most group posterior inclusion probabilities (PIPs) and individual PIPs were low (<0.50), except for BPA (PIP = 0.99) (Table S2). For male infants, most group and individual PIPs were low (<0.50), except for BPA (PIP = 0.58) (Table S3). This indicates that BPA was selected as the most important mixture component with respect to EAA for both female and male infants. Most of the univariate exposure-response functions showed evidence of weak to moderate relationships, except for BPA, which showed a positive cubic association with EAA in female infants (Figure S3) and a positive linear association for male infants (Figure S4). There were overall null effects of EDC mixtures on EAA in female (Figure S5) and male infants (Figure S6).

### 2.6. Supplementary Analyses

In supplementary analyses that examined sex-adjusted associations between EDCs and EAA in the entire sample (n = 224) (Table S4), higher prenatal exposure to BPA was associated with increased EAA (*B* = 0.67, 95% CI: 0.29, 1.03, *p* = 0.001, adjusted *p* = 0.01). This association survived FDR correction. No other EDCs were associated with EAA in the entire sample.

In supplementary analyses that examined sex-specific associations between EDCs and EAA as estimated by the Horvath pan-tissue clock, one finding was replicated for female infants. Higher prenatal exposure to MNP was associated with decreased EAA in this sub-group (*B* = −0.04, 95% CI: −0.07, −0.01, *p* = 0.02, adjusted *p* = 0.06) (Table S5).

## 3. Discussion

Data from a sample of 224 mother–infant pairs enrolled in a Canadian prospective pregnancy cohort (APrON) were used to examine the associations between prenatal exposure to two classes of EDCs, bisphenols and phthalates, and epigenetic age as estimated by the PedBE clock. In female infants, we found that higher maternal concentrations of second trimester BPA were associated with increased EAA (or ‘faster’ biological aging). For male infants, we found indication of a marginal association, in the same direction. Moreover, in female infants, we found that higher maternal concentrations of multiple second trimester phthalate metabolites were associated with decreased EAA (or ‘slower’ biological aging). This was the first examination of prenatal exposure to these chemicals and PedBE EAA. Although these results require replication, the present findings suggest that an epigenetic aging biomarker of these prenatal chemical exposures is detectable in a sex-specific manner in infants as young as 3 months of age.

We used the PedBE clock to estimate DNA methylation-based age, as determined by 94 age-related CpGs, and found an association between prenatal BPA exposure and this epigenetic aging biomarker in female infants. To our knowledge, no prior work has directly examined prenatal exposure to BPA and DNA methylation variation in this tissue. Our finding is consistent with previous research linking gestational exposure to BPA and DNA methylation alterations in cord blood [22,24,34,35]. For instance, an epigenome-wide association study (EWAS) reported associations between maternal urinary BPA concentrations during the first trimester of pregnancy and altered DNA methylation at 38 individual CpGs in cord blood [22]. There is also evidence that epigenomic changes associated with gestational BPA exposure may differ by sex. Another recent epigenome-wide analysis found 28 male- and 16 female-specific differentially methylated CpGs in cord blood that were associated with cord blood concentrations of BPA [24]. They found further evidence of sex-specific associations in their KEGG pathway analysis; for example, that *AMPK* signaling and natural killer cytotoxicity pathways were enriched in males, and estrogen signaling and B cell receptor signaling pathways were enriched in females [24]. Relatedly, a candidate gene study found that maternal first trimester concentrations of BPA were associated with differential cord blood DNA methylation in genes related to growth and development (e.g., *IGF2*, *PPARA*) in females only [34]. Overall, these findings indicate that in utero exposure to BPA can shape epigenomic responses in a sex-specific manner and suggest that through programming important developmental pathways (e.g., immune, hormone signaling) it may have enduring health effects. Further research is needed to characterize the effects of in utero exposure to BPA on neonatal and infant epigenomes, ideally with replication analyses across multiple cohorts to validate results, and to better understand the nature of potential sex-specific effects.

It is possible that the magnitude of the association between prenatal BPA exposure and EAA in infants differs by sex, with a larger positive association in females. We found that female infants displayed faster biological aging following higher prenatal exposure to BPA (*B* = 0.72, adjusted *p* = 0.04). Notably, we found a smaller non-significant effect in male infants (*B* = 0.52, adjusted *p* = 0.54) and a similar effect in our sex-adjusted supplementary analyses (*B* = 0.67, adjusted *p* = 0.01), indicating that an association for males could be found in future research. Further, our mixture analyses revealed that prenatal BPA was positively related to EAA in both female and male infants, but there was a stronger relationship in females. BPA can bind to estrogen receptors and affect sex-specific brain development, with some evidence from human studies and animal research reporting greater effects in females [36,37]. This provides support for our proposition that the association between prenatal BPA and EAA may be larger in females. Overall, these findings are generally consistent with increasing evidence that environmental exposures, such as endocrine disruptors and particle pollution (PM_2.5_), may accelerate biological aging [38]. It is currently unclear what implications faster biological aging may have for infant development. Previous work examining DNA methylation-based age as estimated by the PedBE clock reported that infants born very preterm displayed accelerated PedBE age, compared to neonates born at a later gestational age [39]. They also found that accelerated PedBE age was associated with smaller infant brain volumes and slower brain growth, as well as poorer performance on the Bayley Scales of Infant Development-Third Edition (Bayley-III) cognitive and languages scales at 18 months of age [39]. This suggests that faster epigenetic aging may be related to greater neurodevelopmental risk, and this may be increased by higher prenatal exposure to BPA. This proposition is consistent with the results of previous research using data from this cohort, which found that higher maternal second trimester BPA concentrations were associated with less developed white matter microstructure in two tracts of the brain [40] and sex-specific neurodevelopmental outcomes (i.e., social emotional scores on the Bayley-III at age 2) [17]. To enable future interpretations of the health implications of increased pediatric EAA, as well as to unravel potential sex-specific developmental risk, more studies are needed that examine early environmental factors, epigenetic aging biomarkers, and neurodevelopmental outcomes.

We also found that PedBE DNA methylation-based aging was associated with prenatal phthalate exposure, but this association was specific to female infants. This finding is similar to earlier work using a different sub-sample from this cohort and an older Illumina platform (i.e., 450k); this EWAS identified 12 unique CpGs associated with prenatal exposure to high and/or low molecular weight phthalates in infant buccal epithelial cells and another 12 in venous bloods [23], many of which were loci previously found to predict DNA methylation-related age in individuals aged 6 to 17 years [41]. This identification of prenatal phthalate-associated CpGs in infant tissues is similar to previous epigenome-wide analyses that discovered associations between maternal phthalate concentrations during pregnancy and site-specific DNA methylation alterations in cord blood [19,20,21,42]. However, little is known about potential sex-dependent associations. Several studies have found female-specific CpGs associated with prenatal exposure to some phthalates and metabolites (e.g., di-isononyl phthalate (DiNP), butyl benzyl phthalate (BBzP), MnBP, MCPP, MEPP) [20,34,43]. For example, one candidate gene analysis found that maternal levels of MEP and MCPP during the first trimester of pregnancy were associated with altered cord blood DNA methylation of a growth-related gene (i.e., *IGF2*) and the estrogen receptor 1 gene (*ESR1*), respectively, in females only [34]. These cumulative results suggest that in utero exposure to phthalates can also shape sex-specific epigenetic profiles, but more investigations are needed to verify and validate previous findings.

We found that one metabolite of dimethyl phthalate (i.e., MMP), a secondary metabolite of DiNP (i.e., MCOP), and a metabolite of di-isononyl phthalate (i.e., MNP) were associated with decreased EAA in female infants (*B*’s –0.36 to −0.66, adjusted *p* < 0.10). We can be confident in the association for MCOP (adjusted *p* < 0.05). We are also confident in our finding for MNP as it was replicated in our supplementary analyses that examined Horvath pan-tissue EAA. In contrast to our findings, a recent study reported male-specific associations between prenatal exposure to phthalates and DNA methylation-based age. Using data from 385 maternal–child pairs enrolled in the CHAMACOS cohort, researchers found a negative association between prenatal DEHP and intrinsic EAA estimated by the Horvath pan-tissue clock in males at 7 years of age (*B* = −0.62, adjusted *p* = 0.04), but a non-significant effect in females (*B* = −0.05, adjusted *p* = 0.71) [28]. It is probably unsuitable to directly compare the results of our study to those from the study conducted using the CHAMACOS cohort due to differences in sample characteristics (e.g., 62% of mothers in that CHAMACOS sample were living at or below the poverty line) and study design (e.g., different epigenetic aging biomarkers from different tissues collected at different timepoints). Other prenatal exposures have also been linked to decreased EAA in populations of older children and adolescents. For example, a study using a sub-sample of Avon Longitudinal Study of Parents and Child (ALSPAC) maternal–child pairs, found that maternal concentrations of another endocrine disruptor, cadmium (*B* = −1.61, *p* = 0.05), and cotinine (*B* = −0.001, *p* = 0.05) during pregnancy were associated with decreased average EAA during childhood and adolescence estimated by the Horvath pan-tissue clock [44]. Although the life-course dynamics of epimutations are not well understood, and it is possible that ‘slower’ biological aging as evidenced by decreased EAA may reflect delayed age-related development [27], more basic research is needed to clarify the longitudinal associations between a variety of early environmental factors and variation in biological aging rates. This may enable future interpretation of why certain prenatal chemical exposures, such as bisphenols and phthalates, may be associated with increased versus decreased EAA in pediatric populations.

It is possible that this pediatric-buccal-epigenetic aging biomarker may be picking up on environmental chemical-induced damage that may have implications for children’s health. Biological aging as measured by the PedBE has been associated with clinical phenotypes in children. For example, increased PedBE EAA has been associated with internalizing disorders in children [45], while decreased PedBE EAA, or ‘younger’ biological age, has been reported in children who formerly had a pediatric critical illness requiring intensive care, compared to healthy children, which was correlated with stunted growth in height but not weight [46]. Thus, it is also possible that, whether increased or decreased, some deviations between PedBE age and chronological age may signify risk for behavioral problems and future psychopathology, while others may be indicators of adverse physical health and development outcomes. This may be the case for the present findings, as prenatal exposure to bisphenols and phthalates have been associated with internalizing and externalizing problems in children [15,47,48], as well as anthropometric measurements [49,50,51]. It is important to note that children’s epigenomes have a high degree of malleability, with substantial epigenetic remodeling occurring during the first 5 years of life [52,53]. Thus, it is currently difficult to interpret the potential developmental implications of increased or decreased EAA in the timescale of weeks. There has been limited investigation of the reliability and accuracy of estimating pediatric biological age using DNA methylation values across different tissue types and arrays, but the PedBE clock is deemed the most suitable epigenetic clock for use with pediatrics BECs [54]. Using this tool, we found that each doubling of prenatal BPA was associated with a 0.72 week increase in biological age, and each doubling of several prenatal phthalate metabolites (i.e., MMP, MCOP, MNP) was associated with a 0.36 to 0.66 week decrease in biological age. Although it is tempting to speculate further, we cannot evaluate the precision of PedBE EAA estimates, and this is an important area to be examined by future research. Yet, these findings were in 3-month-old infants, and biological age perturbations on this timescale may reflect meaningful developmental changes; however, large-scale and diverse longitudinal epidemiological investigations are needed to examine whether these effects may diminish or escalate over time. Overall, epigenetic clocks are thought to be informative tools with the potential to provide valuable insight as to whether early interventions mitigate or reverse disease risk, but the application of these tools in toxicology research is still quite novel [55]. There are exciting opportunities for future investigations to examine the potential reversibility of epigenetic changes, particularly those associated with risk for clinical difficulties, through nutritional, pharmacological, or psychosocial interventions delivered in early childhood.

In contrast to populations of adults, where increased EAA has been frequently linked to disease risk and shorter lifespan [56], less is known about biological aging rates in pediatric populations. It is possible that epigenetic age estimates in infants and young children may be strongly connected to growth and developmental trajectories. The CpGs included in the PedBE clock are annotated to genes involved in pathways important for growth, and PedBE EAA was found to be associated with obstetric outcomes, including birthweight [33]. Thus, it is plausible that the PedBE biomarker may represent general developmental patterns of growth that are sensitive to prenatal chemical exposures. Interestingly, although results are quite mixed [51,57,58], one meta-analysis found that prenatal BPA was positively associated with birthweight [59], and it has been suggested that this association may be U-shaped [60]. Multiple meta-analyses have reported that prenatal phthalates were inversely associated with birthweight [49,50], and that these associations were sex-specific [50,61]. Specifically, one recent meta-analysis reported that prenatal exposure to high molecular weight phthalates and MMP was associated with reduced birthweight in female infants [61]. As we found relationships in similar directions with respect to these chemicals (i.e., BPA, MMP, two high molecular weight phthalate metabolites—MCOP and MNP) and EAA, this lends further support to our proposition that PedBE picks up on age-related growth trajectories impacted by environmental exposures. The developmental origins of health and disease (DOHaD) hypothesis emphasizes the role of the early environment in programming later growth and development through epigenetic mechanisms [62,63], and future studies that examine epigenetic aging biomarkers as potential mediators of the associations between EDCs and child behavioral, immune, brain, and physical health outcomes may help to shed light on whether molecular processes related to aging are underlying these associations.

### Strengths and Limitations

This study provides the first characterization of prenatal exposure to two common classes of EDCs on DNA methylation-based aging in infants. Our methods and analyses have some notable strengths. We used the PedBE clock to estimate DNA methylation-based age, as this is the best option for this tissue type and population [27]. We conducted a comprehensive set of analyses that included sex-stratified models, chemical mixture models, supplementary sex-adjusted models, and supplementary models that examined another epigenetic clock recommended for pediatric samples [27]. To examine associations, we used robust regression methods, which are robust to violations of statistical assumptions and produce trustworthy regression estimates [64,65]. We also used FDR to correct for multiple comparisons and control the rate of type I errors (i.e., false positives). This rigorous analytical approach allows us to be more confident that our findings and conclusions are not erroneous.

This study’s findings are subject to several limitations. First, urinary bisphenol and phthalate exposures were only measured once during the second trimester of pregnancy. Urinary concentrations of EDCs show temporal variability [66], but single timepoint sampling has been shown to demonstrate moderate sensitivity for predicting a pregnant woman’s level of exposure [67]. The bisphenol and phthalate concentrations detected in this sample (Table 2 and Table 3) are higher than those reported in another Canadian pregnancy cohort study (e.g., GM of BPA of 0.80 μg/L, GM of MEP 32.02 μg/L, GM of MEOHP of 6.39 μg/L in MIREC) [5], while the bisphenol and low molecular weight phthalate metabolite concentrations are lower than those reported in pregnant women from the Netherlands (e.g., median BPA of 1.66 μg/L, median BPS of 0.36 μg/L, median MEP of 138.03 μg/L in the Generation R study) [68]. Thus, the present results may not be generalizable to other samples with different exposure levels. It is also possible that bisphenol and phthalate exposures during other trimesters of pregnancy and/or the neonatal period may influence EAA in infants. Based on available data, 98.9% of women in this study reported breastfeeding at 3 months postpartum; however, 54.0% of APrON women reported exclusively breastfeeding at 3 months postpartum [69]. Thus, it is possible that postnatal exposure to EDCs such as BPA or DEHP through infant formulas [70] could have influenced the present associations. However, infant urines were not collected from this sample and the potential influence of postnatal exposure to EDCs on infant EAA could not be examined. Future research is needed that examines potential trimester-specific effects and EDC exposures during the first few months of life.

Another limitation of the present study is that our relatively small sample size (n = 224; 54.0% male infants) limited the power of our analyses to detect smaller effects, and some of our results had wide confidence intervals (i.e., overall chemical mixture effects). To our knowledge, this is the first investigation of the associations between prenatal exposure to bisphenols and phthalates and PedBE EAA, and the present results require replication. Future research with larger sample sizes may help to clarify the associations between different EDCs and sex-specific deviations in EAA. It is possible that we did not detect any significant overall effects of the EDC chemical mixture as there were low-to-moderate univariate associations between the individual chemical components and EAA, some of which were in different directions. This is similar to the results of a recent epigenome-wide investigation using data from the Generation R Study, which found no associations between a mixture of bisphenols and phthalates during pregnancy and DNA methylation alterations in cord blood [71]. This may indicate that although chemicals may belong to the same or a similar category (e.g., EDCs) or class (e.g., high molecular weight phthalates), they may not exhibit similar biological effects. This is an important consideration in future analyses of chemical mixture effects. Relatedly, this study is an epidemiological investigation, and it is associative in nature; animal research is needed that examines causal mechanisms. For example, BPA has been shown to affect estrogen receptor signaling [36,37], while phthalates can exert estrogenic and anti-androgenic effects [72,73]. Research using model organisms is needed to determine how EDCs can act simultaneously and potentially differentially on biological mechanisms (e.g., epigenetic regulation of hormonal receptors, epigenetic aging biomarkers), which could help inform future human research in this area. Lastly, due to our relatively low-risk and homogenous sample (i.e., mothers were predominantly White and of high socioeconomic advantage), our findings may not apply to other populations.

## 4. Materials and Methods

### 4.1. Study Sample

Participants included a sample of maternal–child pairs (n = 224) recruited between 2009 and 2012 from the Alberta Pregnancy Outcomes and Nutrition (APrON) study [49,50]. Specifically, participants were from the APrON Neurotox sub-study. Inclusion criteria for the present study were as follows: (i) a maternal non-fasting urine sample provided during the second trimester of pregnancy, (ii) mothers did not report smoking, consuming alcohol, or receiving steroids during pregnancy, and (iii) a buccal epithelial cell (BEC) sample obtained from infants at 3 months of age (see Figure S7 for participant flow diagram). The research protocol was approved by the Conjoint Health Research Ethics Board at the University of Calgary. Written, informed consent was obtained from families prior to the completion of questionnaires and the collection of samples.

### 4.2. Bisphenol and Phthalate Exposures

Maternal non-fasting urine samples were collected during the second trimester of pregnancy (mean = 17 weeks of gestation). Sterile cups were used to collect the samples, which were then aliquoted directly into cryovials, and stored at −80 °C. The methods describing sample collection protocol, quality control experiments, and quantification of total bisphenols at the University of Alberta [6,74] and phthalate metabolites at the Alberta Centre for Toxicology have been previously described [75,76]. The limit of detection (LOD) of total BPA was 0.32 μg/L, while the LODs for total bisphenol S (BPS) and the 12 phthalate metabolites were 0.10 μg/L. Additional information on the individual metabolites quantified and calculation of molar sums of ΣHMWPs, ΣLMWPs, and ΣDEHP is provided (see Supplement). Non-detectable concentrations were given values of LOD/√2 [6,77,78,79]. To account for urinary dilution, urinary creatinine was included as a covariate in models that examined the associations between bisphenol and phthalate exposures and EAA in infants.

### 4.3. DNA Methylation Quantification and Epigenetic Age Estimation

When infants were approximately 3 months of age, BECs were collected using sterile cytology swab brushes. To ensure sufficient sample collection, brushes were rubbed up and down the infant’s entire cheek 10 times on two different swabs. The BECs were kept in short-term storage at −80 °C before DNA extraction, and isolated DNA was stored long-term at 4 °C at the genetics laboratory, Alberta Children’s Hospital. DNA extraction was completed by cell lysis, followed by purification using the Gentra Puregene system (Qiagen, Venlo, Limburg, The Netherlands).

Using the established protocol, DNA methylation values were extracted and processed [23]. In brief, a sample of 750 ng of genomic DNA was used for bisulfite conversion using the EZ DNA Methylation Kit (Zymo Research, Irvine, CA, USA). Following the manufacturer’s protocol, quantitative DNA methylation measurements of purified bisulfite converted DNA were performed using the Infinium MethylationEPIC BeadChip platform (850 K array) [80]. A sample of 160 ng of bisulfite converted DNA was whole genome amplified, fragmented, and hybridized to BeadChip arrays. Each CpG site of the 850K platform is targeted by a probe to distinguish ‘methylated’ and ‘unmethylated’ intensity through different dye colors (i.e., green and red). Methylation levels were calculated by dividing the methylation probe signal intensity by the sum of the methylated and unmethylated probe signal intensities in Illumina GenomeStudio (San Diego, CA, USA).

A normalized, background-corrected, and color-corrected file of methylation levels, as previously described [23], was used to estimate epigenetic age. The PedBE age was estimated using the publicly available online script [81]. The PedBE clock is a validated tool for use with individuals from 0 to 20 years of age that is capable of accurately calculating DNA methylation-based age based on 94 CpG sites [33]. Epigenetic age acceleration (EAA) was defined as the residuals from regressing the estimated DNA methylation-based age on the infants’ actual (i.e., chronological) age correcting for estimated BEC proportion as derived from the EpiDISH package [82]. Thus, EAA provides a measure of whether infants are aging ‘faster’ (i.e., increased EAA) or ‘slower’ (i.e., decreased EAA) biologically than their chronological age. This was the biological aging estimate used in the main analyses. PedBE age difference was also calculated; this was the difference between the estimated epigenetic age and chronological age. In this sample, PedBE EAA and age difference were synonymous as the children’s age range at sample collection was very small (*rho* = 0.97, *p* < 0.001).

In supplementary analyses, epigenetic age was estimated using the Horvath pan-tissue epigenetic clock using the online age calculator [29]. The Horvath pan-tissue clock is an estimator of DNA methylation-based age based on 353 CpG sites. EAAs as estimated by the PedBE and the Horvath pan-tissue epigenetic clock were only moderated correlated (*rho* = 0.31, *p* < 0.001).

### 4.4. Covariates

This study selected potential covariates based on variables previously identified to be associated with prenatal exposure to bisphenols and phthalates and/or epigenetic modifications [23,24,25,28,83]. Potential covariates included the following maternal characteristics: self-reported race, education, marital status, household income, age, parity, and pre-pregnancy body mass index (BMI). Potential covariates also included the following child characteristics: gestational age at birth and birthweight. Dichotomous variables were coded as follows: maternal self-reported race (i.e., White versus not White), maternal education (i.e., undergraduate university degree or higher versus trade school/high school diploma/lower), maternal marital status (i.e., married/common-law versus single/separated/divorced), household income (i.e., less than $70,000 Canadian dollars versus greater than or equal to $70,000 Canadian dollars), maternal parity (i.e., primiparous versus multiparous). Breastfeeding was another potential covariate, but it was not included in the present analysis; based on available data, 98.9% of the women were breastfeeding at 3 months postpartum. Urinary creatinine was included in all models to account for urine dilution. Infant age at sample collection was included as a covariate in all models given the influence of sample collection timepoint on EAA [84].

### 4.5. Statistical Analyses

Analyses were conducted in version 4.3.0 of R. To inspect differences in sample characteristics between sub-groups stratified by infant sex, analyses of variance (ANOVAs) and chi-squared (χ^2^) tests were used.

To examine associations between EDCs and EAA in infants, robust multivariable linear regressions were performed using the *MASS* package and a Huber M-estimator [64,85]. This approach was used as it can adapt to skewed distributions and provide reliable estimates [86,87]. Robust methods are preferable to other common methods (e.g., OLS regression) as they are less sensitive to the effects of influential observations, and accommodate non-normality, heteroscedasticity, and non-linearity [86]. In all models, log2-transformed concentrations of bisphenols and phthalates were used. This is a transformation commonly applied to gestational concentrations of these chemicals for interpretative purposes [88,89,90], as this means that regression coefficients (*B’s*) are expressed per doubling of chemical concentration. Covariates were included in models if they were associated with EAA at *p* < 0.20 [91]. The following covariates were included in the models for female infants: second trimester urinary creatinine, maternal education, maternal marital status, maternal age, infant gestational age at birth, infant birthweight, and infant age at sample collection. The following covariates were included in models for male infants: second trimester urinary creatinine, maternal marital status, annual household income, maternal age, maternal pre-pregnancy BMI, infant gestational age at birth, infant birthweight, and infant age at sample collection. A directed acyclic graph was constructed to illustrate the hypothesized relationships between variables (Figure S8).

We corrected for multiple comparisons using the *qvalue* package [92] to implement the Benjamini–Hochberg procedure to control the false discovery rate (FDR; i.e., proportion of false positives) [93]. We report *q*-values (i.e., adjusted *p*-values) from 0.05 to 0.10, which yield FDRs of 5% and 10%, respectively. We have the most statistical confidence in results at *q* < 0.05.

In addition to the main analyses, chemical mixture effects were examined using Bayesian kernel machine regression (BKMR) [94] using the *bkmr* package [95]. BKMR models estimated the individual and overall effects of the 14 target analytes (2 bisphenols and 12 phthalate metabolites) on EAA for female infants (n = 103) and male infants (n = 118; one twin from three sets of twins was removed due to non-independence of mixtures). BKMR produces posterior inclusion probabilities (PIPs), which represent the probability that each EDC exposure was selected for inclusion (20,000 iterations completed).

Supplementary analyses examined the sex-adjusted associations between EDCs and EAA in the overall sample. Associations between EDCs with another epigenetic aging measure, EAA estimated by the Horvath pan-tissue epigenetic clock, were examined in the sex-stratified groups.

## 5. Conclusions

We are only beginning to understand deviations between estimated epigenetic age and chronological age in young children, and that DNA methylation-based aging is not a linear process across the lifespan [96]. The present results suggest that two classes of EDCs, bisphenols and phthalates, may have divergent effects on DNA methylation-based age estimated in pediatric buccal cells. This may indicate that early exposure to these EDCs is associated with different molecular processes that ‘get under the skin’ and influence epigenetic aging biomarkers in young children. Continued epigenetic investigations of early chemical exposures and child health outcomes are needed to help understand how early environmental insults may alter age-related developmental trajectories and confer risk.

## Figures and Tables

**Table 1:** Participant characteristics for the entire sample and the sub-groups stratified by infant sex.

	Entire Sample (n = 224)	Female Infants (n = 103)	Male Infants (n = 121)	*p*-Value ^1^
Characteristic	N (%); M ^2^ (SD ^3^, Range)	N (%); M (SD, Range)	N (%); M (SD, Range)	
Maternal self-reported race				0.8
White	189 (84.4%)	88 (85.4%)	101 (83.5%)	
Non-White	35 (15.6%)	15 (14.6%)	20 (16.5%)	
Maternal education				0.6
University/postgraduate training/graduate school	163 (72.8%)	77 (74.8%)	86 (71.1%)	
High school/technical school/trade school/college	61 (27.2%)	26 (25.2%)	35 (28.9%)	
Maternal marital status				0.7
Married/common-law	215 (96.0%)	100 (97.1%)	115 (95.0%)	
Single/separated/divorced	9 (4.0%)	3 (2.9%)	6 (5.0%)	
Annual household income				0.3
≥70,000 Canadian dollars	183 (81.7%)	88 (85.4%)	95 (78.5%)	
<70,000 Canadian dollars	41 (18.3%)	15 (14.6%)	26 (21.5%)	
Maternal parity				0.01 *
Primiparous	129 (57.6%)	49 (47.6%)	80 (66.1%)	
Multiparous	95 (42.4%)	54 (52.4%)	41 (33.9%)	
Maternal age (years)	32.0 (4.3)	32.4 (3.9)	31.7 (4.6)	0.2
Maternal pre-pregnancy BMI ^4^	24.7 (4.9)	24.8 (5.0)	24.6 (4.8)	0.8
Gestational age at birth (weeks)	39.0 (1.8)	39.2 (1.8)	38.9 (1.7)	0.2
Infant birthweight (g)	3368 (541)	3332 (519)	3389 (560)	0.4
Infant age at sample collection (weeks)	13.1 (3.1)	12.8 (1.9)	13.3 (3.8)	0.2

* *p* < 0.05. ^1^ *p*-values for F statistics from one-way analyses of variance (ANOVAs) and test statistics (χ^2^) from chi-squared tests for differences between the sex-stratified groups on continuous and categorical variables. ^2^ M = mean. ^3^ SD = standard deviation. ^4^ BMI = body mass index (kg/m^2^).

**Table 2:** Maternal bisphenol, phthalate molar sums, and phthalate metabolite concentrations in second trimester urine samples for the sub-group with female infants (n = 103).

Chemical	% >LOD ^1^	Minimum	Maximum	Geometric Mean	25th Percentile	50th Percentile	75th Percentile
Bisphenols (μg/L)							
BPA	96.1%	<LOD	44.0	1.59	0.801	1.22	2.64
BPS	61.2%	<LOD	243	0.179	0.0707	0.133	0.300
Phthalate Molar Sums (μmol/L)							
ΣLMWPs	-	0.0312	35.6	0.471	0.232	0.429	0.977
ΣHMWPs	-	0.0163	2.96	0.191	0.109	0.210	0.367
ΣDEHP	-	0.00889	1.94	0.0909	0.0378	0.0100	0.168
Phthalate Metabolites (μg/L)							
MMP ^2^	99.0%	<LOD	86.1	2.26	1.31	2.32	4.33
MEP ^2^	100%	1.47	6830	48.4	18.9	41.5	113
MiBP ^2^	98.1%	<LOD	49.3	9.43	5.73	10.4	18.6
MBP ^2^	100%	1.20	157	17.3	9.34	17.6	30.8
MEHP ^3,4^	99.0%	<LOD	120	4.01	1.73	4.73	8.46
MEHHP ^3,4^	100%	1.12	250	12.1	5.13	13.5	22.9
MECPP ^3,4^	100%	1.86	342	16.6	8.18	16.9	34.9
MEOHP ^3,4^	100%	0.901	192	9.89	4.51	10.4	19.0
MBzP ^4^	100%	0.279	187	7.21	3.26	8.02	16.0
MCOP ^4^	100%	0.636	434	13.1	6.67	12.9	25.8
MCNP ^4^	92.2%	<LOD	25.3	1.04	0.542	1.08	2.42
MNP ^4^	97.1%	<LOD	801	3.84	1.52	3.29	8.54

^1^ LOD = limit of detection; 0.32 μg/L for BPA, 0.10 μg/L for BPS and phthalate metabolites. ^2^ phthalate metabolites included in the molar sum of low molecular weight phthalates. ^3^ phthalate metabolites included in the molar sum of DEHP. ^4^ phthalate metabolites included in the molar sum of high molecular weight phthalates.

**Table 3:** Maternal bisphenol, phthalate molar sums, and phthalate metabolite concentrations in second trimester urine samples for the sub-group with male infants (n = 121).

Chemical	% >LOD ^1^	Minimum	Maximum	Geometric Mean	25th Percentile	50th Percentile	75th Percentile
Bisphenols (μg/L)							
BPA	91.8%	<LOD	37.8	1.21	0.517	1.19	2.40
BPS	53.7%	<LOD	10.8	0.155	0.0707	0.0964	0.259
Phthalate Molar Sums (μmol/L)							
ΣLMWPs	-	0.0351	23.8	0.615	0.252	0.502	1.49
ΣHMWPs	-	0.00822	1.18	0.160	0.109	0.166	0.290
ΣDEHP	-	0.00365	0.692	0.0733	0.0476	0.0780	0.132
Phthalate Metabolites (μg/L)							
MMP ^2^	100%	0.231	81.3	2.31	1.43	2.42	3.78
MEP ^2^	100%	1.78	4510	68.3	18.8	53.8	232
MiBP ^2^	98.3%	<LOD	121	9.69	5.83	11.0	19.4
MBP ^2^	100%	1.37	745	16.6	8.97	16.6	29.8
MEHP ^3,4^	98.3%	<LOD	35.7	3.03	1.77	3.37	5.63
MEHHP ^3,4^	100%	0.538	107	9.88	6.35	10.4	16.5
MECPP ^3,4^	100%	0.912	118	14.0	8.62	14.9	25.5
MEOHP ^3,4^	100%	0.458	78.5	8.08	4.85	8.62	14.4
MBzP ^4^	100%	0.355	110	7.61	4.30	7.68	17.0
MCOP ^4^	99.2%	<LOD	730	11.2	4.86	11.9	19.4
MCNP ^4^	88.4%	<LOD	123	0.923	0.434	1.09	1.79
MNP ^4^	98.3%	<LOD	753	3.21	1.26	2.58	7.05

^1^ LOD = limit of detection; 0.32 μg/L for BPA, 0.10 μg/L for BPS and phthalate metabolites. ^2^ phthalate metabolites included in the molar sum of low molecular weight phthalates. ^3^ phthalate metabolites included in the molar sum of DEHP. ^4^ phthalate metabolites included in the molar sum of high molecular weight phthalates.

**Table 4:** Sex-stratified robust regression models for the associations between log2-transformed prenatal exposure to bisphenols and phthalates and infant epigenetic age acceleration (EAA) as estimated by the Pediatric-Buccal-Epigenetic (PedBE) clock.

	Female Infants (n = 103)	Male Infants (n = 121)
Chemical	PedBE EAA *B* (95% CI)	PedBE EAA *B* (95% CI)
Bisphenols (μg/L)		
BPA	0.72 (0.21, 1.24) **	0.52 (−0.06, 1.10)
BPS	0.03 (−0.56, 0.61)	−0.14 (−0.73, 0.45)
Phthalate Molar Sums (μmol/L)		
ΣLMWPs	−0.33 (−0.87, 0.22)	−0.10 (−0.58, 0.38)
ΣHMWPs	−0.27 (−0.93, 0.39)	0.40 (−0.42, 1.22)
ΣDEHP	−0.45 (−1.03, 0.13)	0.37 (−0.38, 1.13)
Phthalate Metabolites (μg/L)		
MMP ^1^	−0.66 (−1.28, −0.04) *	0.37 (−0.32, 1.06)
MEP ^1^	−0.20 (−0.59, 0.19)	−0.12 (−0.48, 0.24)
MiBP ^1^	−0.57 (−1.31, 0.18)	0.49 (−0.19, 1.17)
MBP ^1^	−0.36 (−1.09, 0.38)	0.12 (−0.63, 0.88)
MEHP ^2,3^	−0.42 (−0.91, 0.07)	0.37 (−0.23, 0.96)
MEHHP ^2,3^	−0.43 (−0.98, 0.13)	0.38 (−0.38, 1.14)
MECPP ^2,3^	−0.42 (−1.04, 0.21)	0.35 (−0.42, 1.12)
MEOHP ^2,3^	−0.46 (−1.06, 0.14)	0.32 (−0.48, 1.11)
MBzP ^3^	0.04 (−0.56, 0.65)	0.10 (−0.57, 0.76)
MCOP ^3^	−0.55 (−1.01, −0.08) **	0.37 (−0.16, 0.90)
MCNP ^3^	−0.28 (−0.77, 0.21)	−0.03 (−0.50, 0.44)
MNP ^3^	−0.36 (−0.69, −0.02) *	0.36 (−0.05, 0.77)

** q ≤ 0.05. * q < 0.10. ^1^ phthalate metabolites included in the molar sum of low molecular weight phthalates. ^2^ phthalate metabolites included in the molar sum of DEHP. ^3^ phthalate metabolites included in the molar sum of high molecular weight phthalates.

## Data Availability

Data are contained within the article and Supplementary Materials.

## Supplementary Materials

The following supporting information can be downloaded at: https://www.mdpi.com/article/10.3390/epigenomes8030031/s1, Figure S1: Histogram with kernel density plot of epigenetic age acceleration values estimated from the Pediatric-Buccal-Epigenetic (PedBE) clock for the entire sample (n = 224). The dashed line displays the mean, which is centered at approximately zero; Figure S2: Kernel density plot of epigenetic age acceleration values estimated from the Pediatric-Buccal-Epigenetic (PedBE) clock for female infants (green) and male infants (purple). The dashed lines display the means for female infants (green line) and for male infants (purple line); Figure S3: Univariate exposure-response functions of log2-transformed prenatal bisphenol and phthalate exposures and epigenetic age acceleration in female infants (n = 103). Associations between each exposure and EAA are plotted while fixing the other exposures at their 50th percentile (95% CIs are shown in grey); Figure S4: Univariate exposure-response functions of log2-transformed prenatal bisphenol and phthalate exposures and epigenetic age acceleration in male infants (n = 118). Associations between each exposure and EAA are plotted while fixing the other exposures at their 50th percentile (95% CIs are shown in grey); Figure S5: Overall effects (95% CIs) of the mixture of endocrine-disrupting chemicals on EAA in female infants (n = 103) using Bayesian kernel machine regression (BKMR) when all the exposures in the mixture at specific percentiles were compared to all the exposures at their 50th percentile; Figure S6: Overall effects (95% CIs) of the mixture of endocrine-disrupting chemicals on EAA in male infants (n = 118) using Bayesian kernel machine regression (BKMR) when all the exposures in the mixture at specific percentiles were compared to all the exposures at their 50th percentile; Figure S7: Participant flow diagram; Figure S8: Directed acyclic graph (DAG) to depict the prior knowledge about pathways related to the research questions. The solid lines illustrate the links between variables included in the models. The dashed line indicates a possible indirect link (not tested); Table S1: Geometric means (GMs) of the bisphenol, phthalate molar sums, and phthalate metabolites in maternal second trimester urine samples for the sub-groups stratified by infant sex; Table S2: Posterior inclusion probabilities (PIPs) for inclusion in the EAA model for female infants (n = 103) using Bayesian kernel machine regression (BKMR); Table S3: Posterior inclusion probabilities (PIPs) for inclusion in the EAA model for male infants (n = 118) using Bayesian kernel machine regression (BKMR); Table S4: Robust regression models, adjusted for infant sex, examining the associations between log2-transformed prenatal bisphenol and phthalate exposures and infant epigenetic age acceleration (EAA) as estimated by the Pediatric-Buccal-Epigenetic (PedBE) clock in the entire sample (n = 224); Table S5: Sex-stratified robust regression models examining the associations between log2-transformed prenatal bisphenol and phthalate exposures and infant epigenetic age acceleration (EAA) as estimated by the Horvath pan-tissue epigenetic clock.
