# Supplementary material for: Sex-Specific Associations between Prenatal Exposure to Bisphenols and Phthalates and Infant Epigenetic Age Acceleration
Source: Epigenomes. 2024 Aug 10;8(3):31. doi: 10.3390/epigenomes8030031 (PMC11348373; doi:10.3390/epigenomes8030031)
Supplement: Supplementary file 1 [file epigenomes-08-00031-s001.zip › epigenomes-3071308-supplementary_FINAL.pdf]

Supplement

Sex-Specific Associations between Prenatal Exposure to Bisphenols and Phthalates and Infant Epigenetic Age Acceleration

Gillian England-Mason [1,2], Sarah M. Merrill [3,4,5], Jiaying Liu [6], Jonathan W. Martin [7], Amy M. MacDonald [8], David W. Kinniburgh [6,8], Nicole Gladish [4,5], Julia L. MacIsaac [4,5], Gerald F. Giesbrecht [1,2,9,10], Nicole Letourneau [1,2,10,11,12,13] Michael S. Kobor [4,5,14], Deborah Dewey [1,2,11,13*]

1 Department of Pediatrics, Cumming School of Medicine, University of Calgary, Calgary, Alberta, T2N 1N4, Canada
2 Owerko Centre, Alberta Children's Hospital Research Institute, University of Calgary, Calgary, Alberta, T2N 1N4, Canada
3 Department of Psychiatry and Human Behavior, The Warren Alpert Medical School at Brown University, Providence, RI, 02903, USA
4 Department of Medical Genetics, British Columbia Children's Hospital Research Institute, University of British Columbia, V6T 1Z4, Vancouver, British Columbia, Canada
5 Centre for Molecular Medicine and Therapeutics, Vancouver, British Columbia, V6H 0B3, Canada
6 Department of Laboratory Medicine and Pathology, University of Alberta, Edmonton, Alberta, T6G 2R3, Canada
7 Science for Life Laboratory, Department of Environmental Science, Stockholm University, Stockholm, 114 19, Sweden
8 Alberta Centre for Toxicology, University of Calgary, Calgary, Alberta, T2N 1N4, Canada
9 Department of Psychology, Faculty of Arts, University of Calgary, Calgary, Alberta, T2N 1N4, Canada
10 Department of Community Health Sciences, Cumming School of Medicine, University of Calgary, Calgary, Alberta, T2N 1N4, Canada
11 Faculty of Nursing, University of Calgary, Calgary, Alberta, T2N 1N4, Canada
12 Department of Psychiatry, Cumming School of Medicine, University of Calgary, Calgary, Alberta, T2N 1N4, Canada
13 Hotchkiss Brain Institute, Calgary, Alberta, T2N 4N1, Canada
14 Program in Child and Brain Development, Canadian Institute for Advanced Research (CIFAR), Toronto, Ontario, M5G 1M1, Canada

Supplemental Materials

Quantification of Urinary Bisphenols and Phthalate Metabolites

During the second trimester of pregnancy (mean = 17.0 weeks of gestation), women provided non-fasting urine samples. Using methods previously described, samples were quantitatively analyzed for total bisphenol A (BPA) and total bisphenol S (BPS) concentrations at the University of Alberta [1,2]. Sterile cups were used to collect the samples, which were then aliquoted directly into cryovials, and stored at -80°C. For sample preparation, aliquots of urine (400 µL) were spiked with 100 ng/mL isotope-labeled BPA (10 µL) and mixed with 1 M ammonium acetate (200 µL), which contained 1 µL of β-glucuronidase and sulfatase aqueous solution (≥ 100,000 units/mL; Sigma, St. Louis, MO) for deconjugation of phase II metabolites. These were incubated overnight at 37°C, and then 1 M formic acid (390 µL) was added to achieve a final volume (1 mL) for analysis. Potential contamination was assessed in field blanks (n = 20) using liquid chromatography-grade water, but no contamination was detected above laboratory blanks. Total urinary BPA and total urinary BPS concentrations were quantified using the Orbitrap Elite analyzer (Thermo Fisher Scientific, California, USA) to perform online solid-phase extraction (SPE) in conjunction with high-performance liquid chromatography (LC)-

high resolution mass spectrometry (MS). The limits of detection (LODs) of total BPA and total BPS were 0.32 and 0.10 μg/L, respectively. Urinary creatinine was analyzed in corresponding aliquots.

Maternal non-fasting urine samples were also quantitatively analyzed for 12 phthalate metabolites. A per previous protocol, this analysis was conducted at the Alberta Centre for Toxicology [3,4]. The 12 phthalate metabolites quantified were: mono-methyl phthalate (MMP), mono-ethyl phthalate (MEP), mono-n-butyl phthalate (MBP), mono-isobutyl phthalate (MiBP), mono-benzyl phthalate (MBzP), mono (2-ethylhexyl) phthalate (MEHP), mono (2-ethyl-5-hydroxyhexyl) phthalate (MEHHP), mono (2-ethyl-5-carboxypentyl) phthalate (MECPP), mono (2-ethyl-5-oxohexyl) phthalate (MEOHP), mono-carboxy-isooctyl phthalate (MCOP), mono-carboxy-isononyl phthalate (MCNP), mono-isononyl phthalate (MNP), mono-cyclohexyl phthalate (MCHP), and mono-n-octyl phthalate (MOP). Two metabolites, MCHP and MOP, were excluded from the statistical analysis due to low detection frequency (< 50%). The metabolites were separated on a 100 x 2.1 mm BetaSil Phenyl Column (Thermo Scientific, Burlington, Ontario, Canada) using an Agilent 1200 HPLC system (Agilent Technologies, LabX, Mississauga, Ontario, Canada). This separation was conducted using a constant column temperature (40°C) and injection volume (10 μL). The metabolites were detected using the QTRAP 5500 system (AB Sciex, Concord, Ontario, Canada). Metabolites were identified using negative multiple reaction

monitoring (MRM; i.e., two MRM transitions at the correct retention time). All metabolites had LODs of 0.10 µg/L. Four metabolites, MEHP, MEHHP, MECPP, and MEOHP were grouped together as the molar sum of di(2-ethylhexyl) phthalate (ΣDEHP); these four metabolites and four additional metabolites, MBzP, MCOP, MCNP, and MNP, were grouped together as the molar sum of high molecular weight phthalates (ΣHMWPs). Four metabolites, MMP, MEP, MBP, and MiBP were grouped together as the molar sum of low molecular weight phthalates (ΣLMWPs).

References (for Supplement)

1. Liu, J.; Wattar, N.; Field, C.J.; Dinu, I.; Dewey, D.; Martin, J.W.; APrON study team Exposure and Dietary Sources of Bisphenol A (BPA) and BPA-Alternatives among Mothers in the APrON Cohort Study. *Environ Int* **2018**, *119*, 319–326, doi:10.1016/j.envint.2018.07.001.
2. England-Mason, G.; Liu, J.; Martin, J.W.; Giesbrecht, G.F.; Letourneau, N.; Dewey, D.; APrON Study Team Postnatal BPA Is Associated with Increasing Executive Function Difficulties in Preschool Children. *Pediatr Res* **2021**, *89*, 686–693, doi:10.1038/s41390-020-0922-6.
3. England-Mason, G.; Merrill, S.M.; Gladish, N.; Moore, S.R.; Giesbrecht, G.F.; Letourneau, N.; MacIsaac, J.L.; MacDonald, A.M.; Kinniburgh, D.W.; Ponsonby, A.-L.; et al. Prenatal Exposure to Phthalates and Peripheral Blood and Buccal Epithelial DNA Methylation in Infants: An Epigenome-Wide Association Study. *Environ Int* **2022**, *163*, 107183, doi:10.1016/j.envint.2022.107183.
4. Soomro, M.H.; England-Mason, G.; Liu, J.; Reardon, A.J.F.; MacDonald, A.M.; Kinniburgh, D.W.; Martin, J.W.; Dewey, D.; APrON Study Team Associations between the Chemical Exposome and Pregnancy Induced Hypertension. *Environ Res* **2023**, *237*, 116838, doi:10.1016/j.envres.2023.116838.

Figure S1. Histogram with kernel density plot of epigenetic age acceleration values estimated from the Pediatric-Buccal-Epigenetic (PedBE) clock for the entire sample (n = 224). The dashed line displays the mean, which is centered at approximately zero.

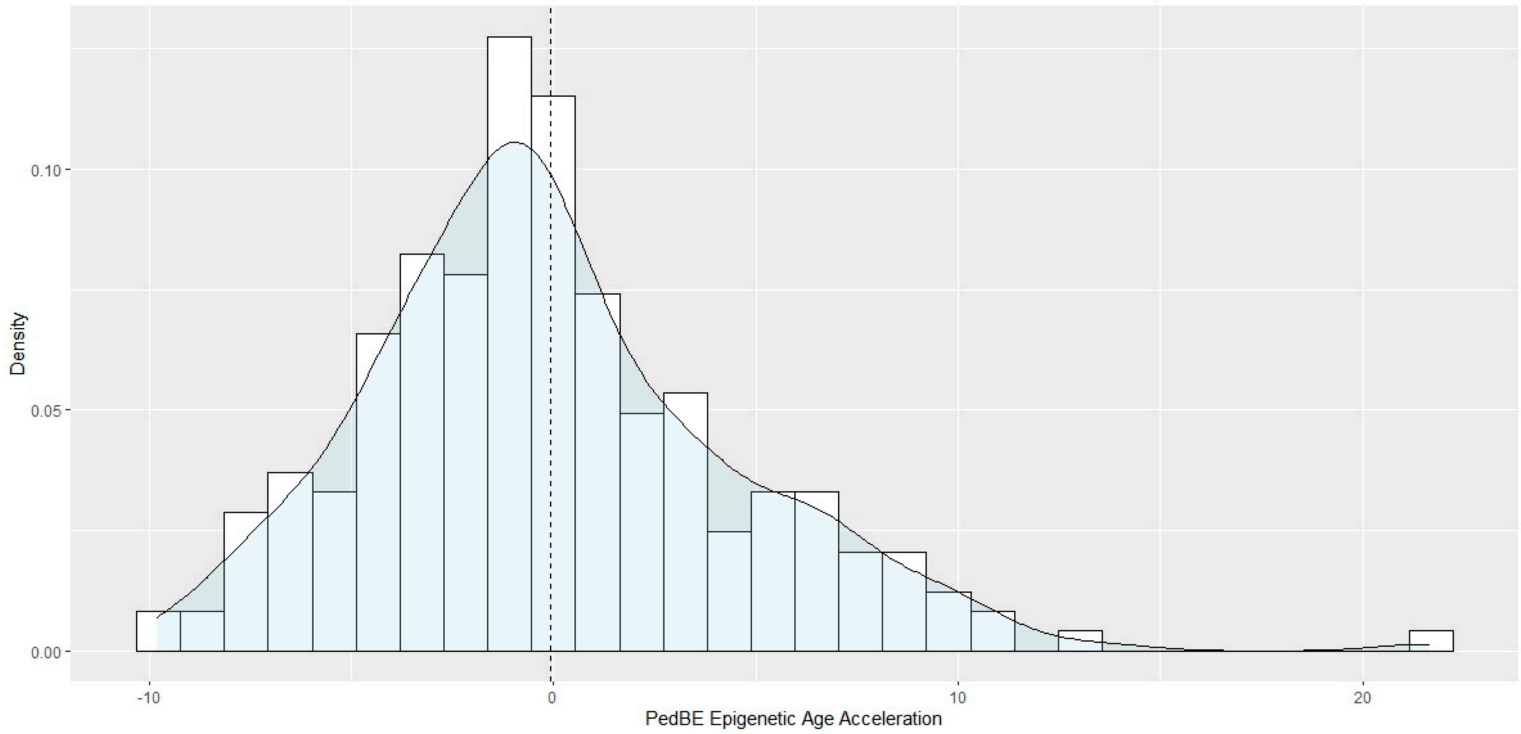

Figure S2. Kernel density plot of epigenetic age acceleration values estimated from the Pediatric-Buccal-Epigenetic (PedBE) clock for female infants (green) and male infants (purple). The dashed lines display the means for female infants (green line) and for male infants (purple line).

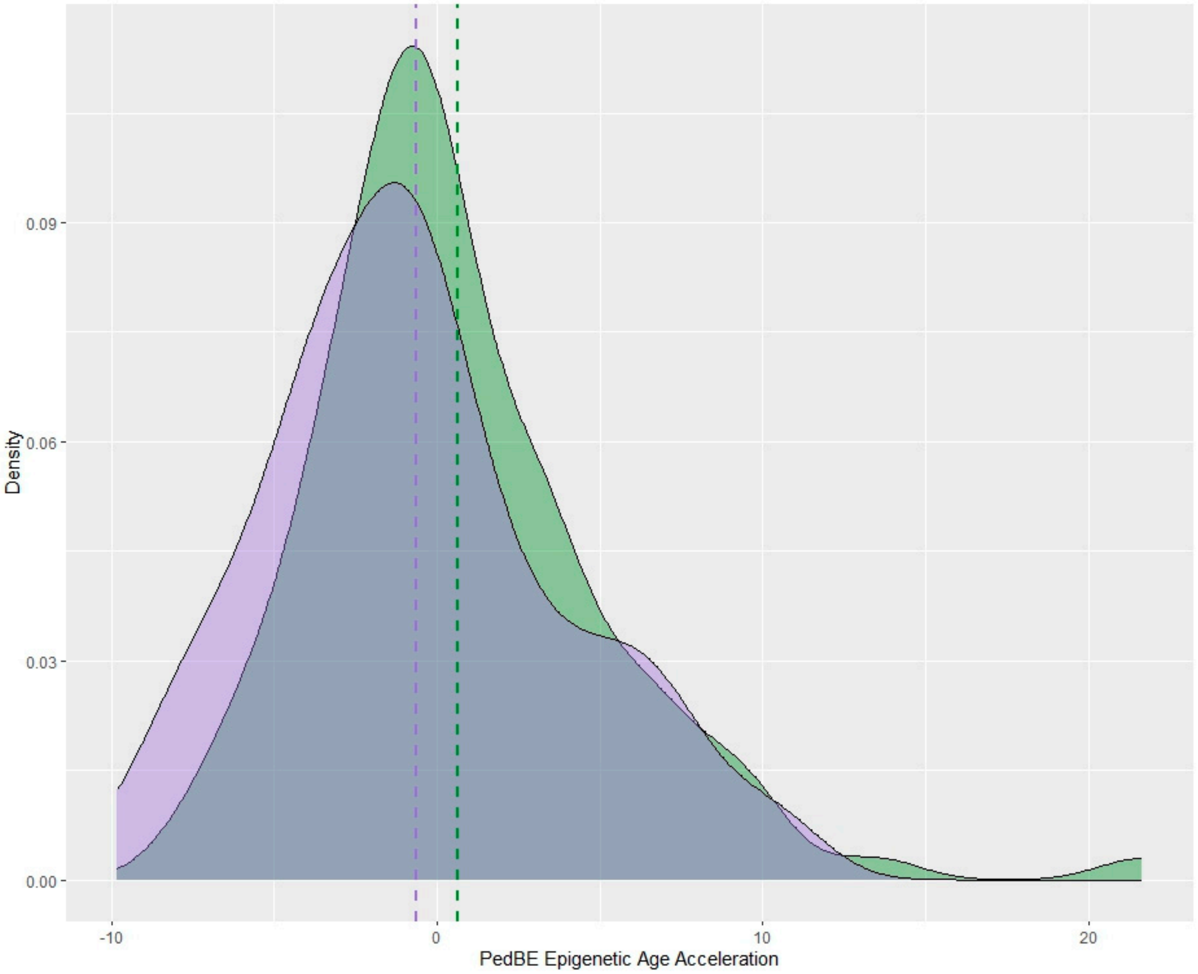

Figure S3. Univariate exposure-response functions of log2-transformed prenatal bisphenol and phthalate exposures and epigenetic age acceleration in female infants (n = 103). Associations between each exposure and EAA are plotted while fixing the other exposures at their 50th percentile (95% CIs are shown in grey).

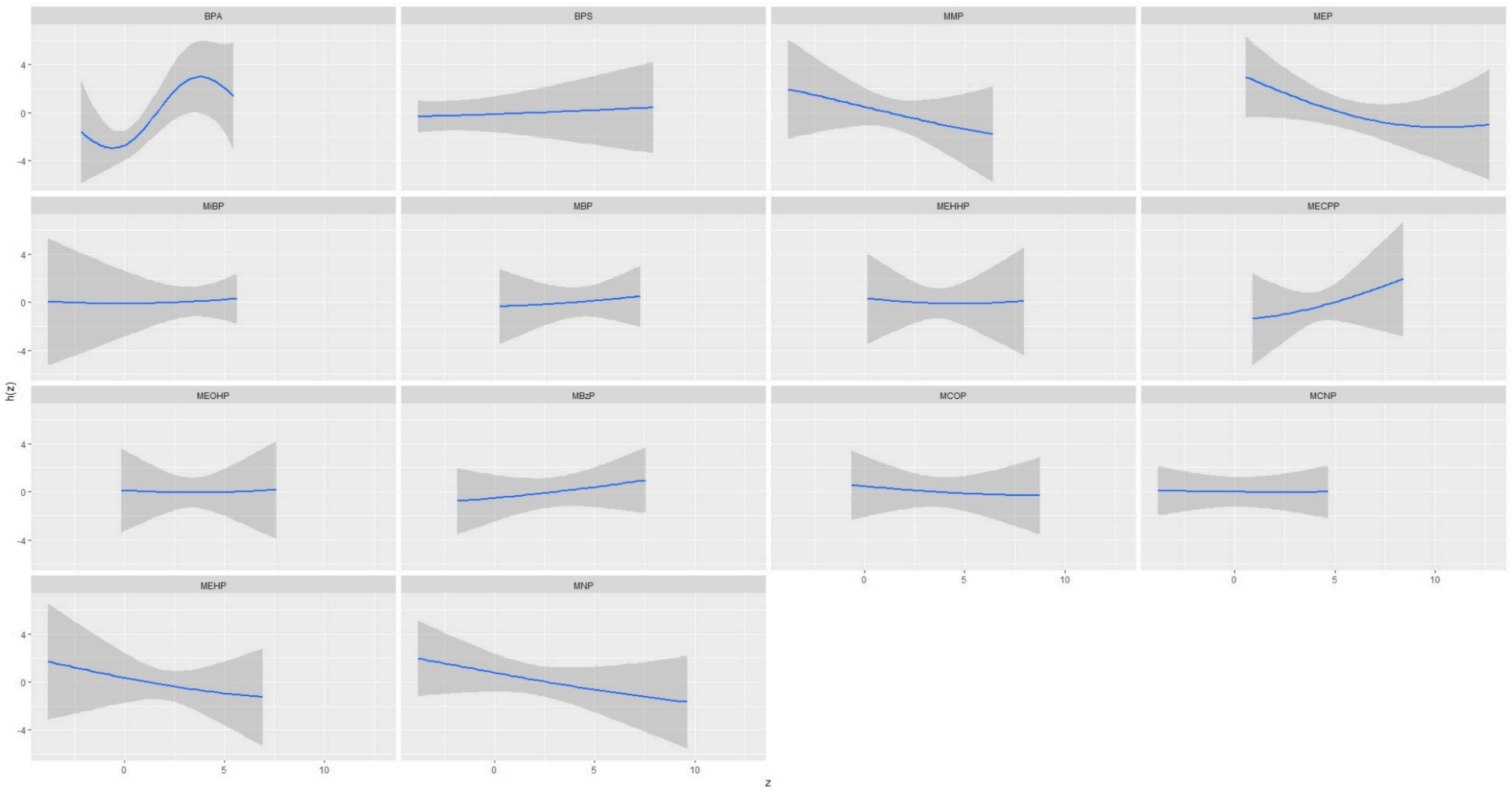

Note. BPA = bisphenol A; BPS = bisphenol S; MMP = mono-methyl phthalate; MEP = mono-ethyl phthalate; MiBP = mono-isobutyl phthalate; MBP = mono-n-butyl phthalate; MEHHP = mono (2-ethyl-5-hydroxyhexyl) phthalate; MECPP = mono (2-ethyl-5-carboxypentyl) phthalate; MEOHP = mono (2-ethyl-5-oxohexyl) phthalate; MBzP = mono-benzyl phthalate; MCOP = mono-carboxy-isooctyl phthalate; MCNP = mono-carboxy-isononyl phthalate; MEHP = mono (2-ethylhexyl) phthalate; MNP = mono-isononyl phthalate

Figure S4. Univariate exposure-response functions of log2-transformed prenatal bisphenol and phthalate exposures and epigenetic age acceleration in male infants (n = 118). Associations between each exposure and EAA are plotted while fixing the other exposures at their 50th percentile (95% CIs are shown in grey).

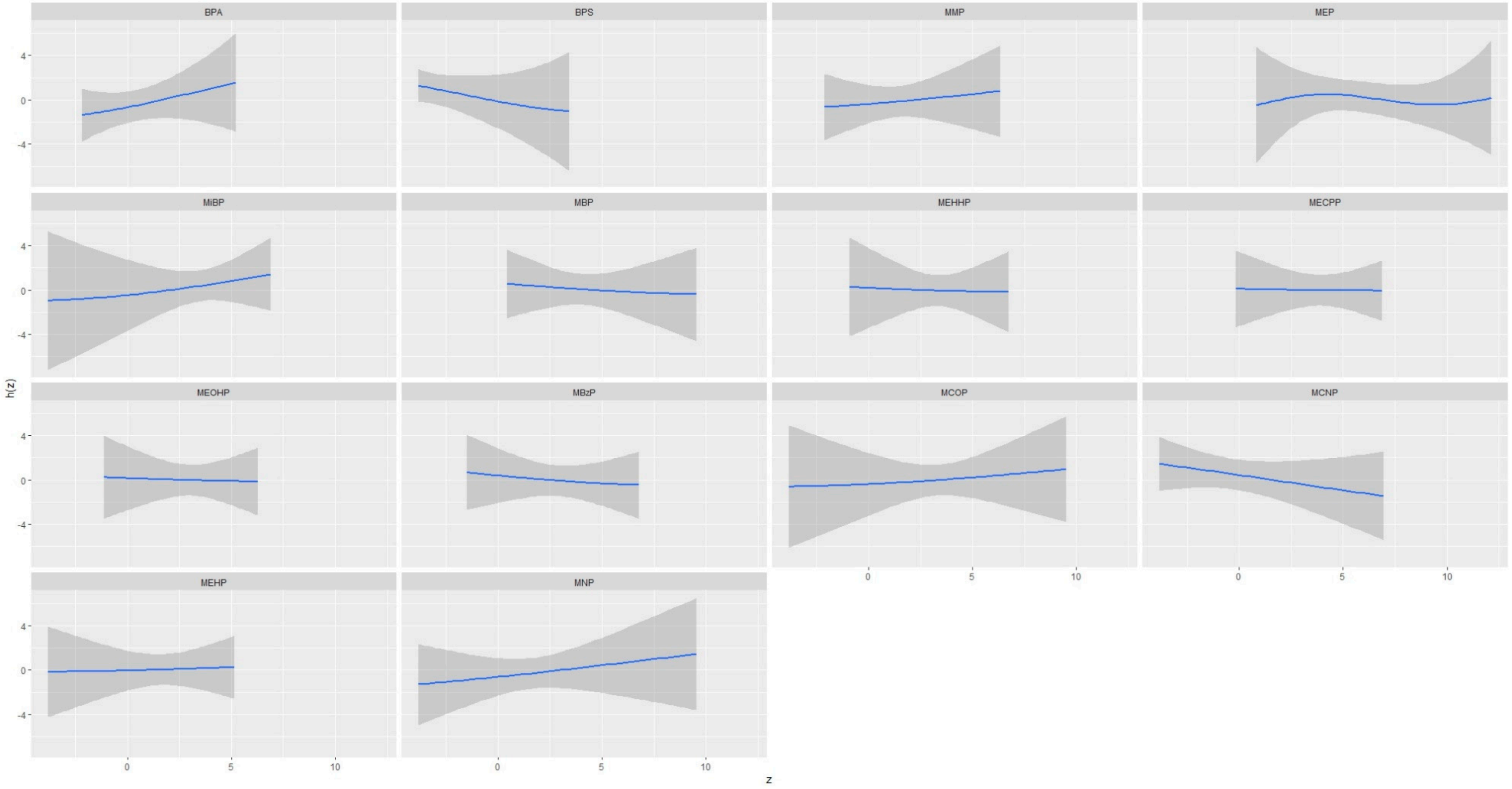

Note. BPA = bisphenol A; BPS = bisphenol S; MMP = mono-methyl phthalate; MEP = mono-ethyl phthalate; MiBP = mono-isobutyl phthalate; MBP = mono-n-butyl phthalate; MEHHP = mono (2-ethyl-5-hydroxyhexyl) phthalate; MECPP = mono (2-ethyl-5-carboxypentyl) phthalate; MEOHP = mono (2-ethyl-5-oxohexyl) phthalate; MBzP = mono-benzyl phthalate;

MCOP = mono-carboxy-isooctyl phthalate; MCNP = mono-carboxy-isononyl phthalate; MEHP = mono (2-ethylhexyl) phthalate; MNP = mono-isononyl phthalate

Figure S5. Overall effects (95% CIs) of the mixture of endocrine-disrupting chemicals on EAA in female infants (n = 103) using Bayesian kernel machine regression (BKMR) when all the exposures in the mixture at specific percentiles were compared to all the exposures at their 50th percentile.

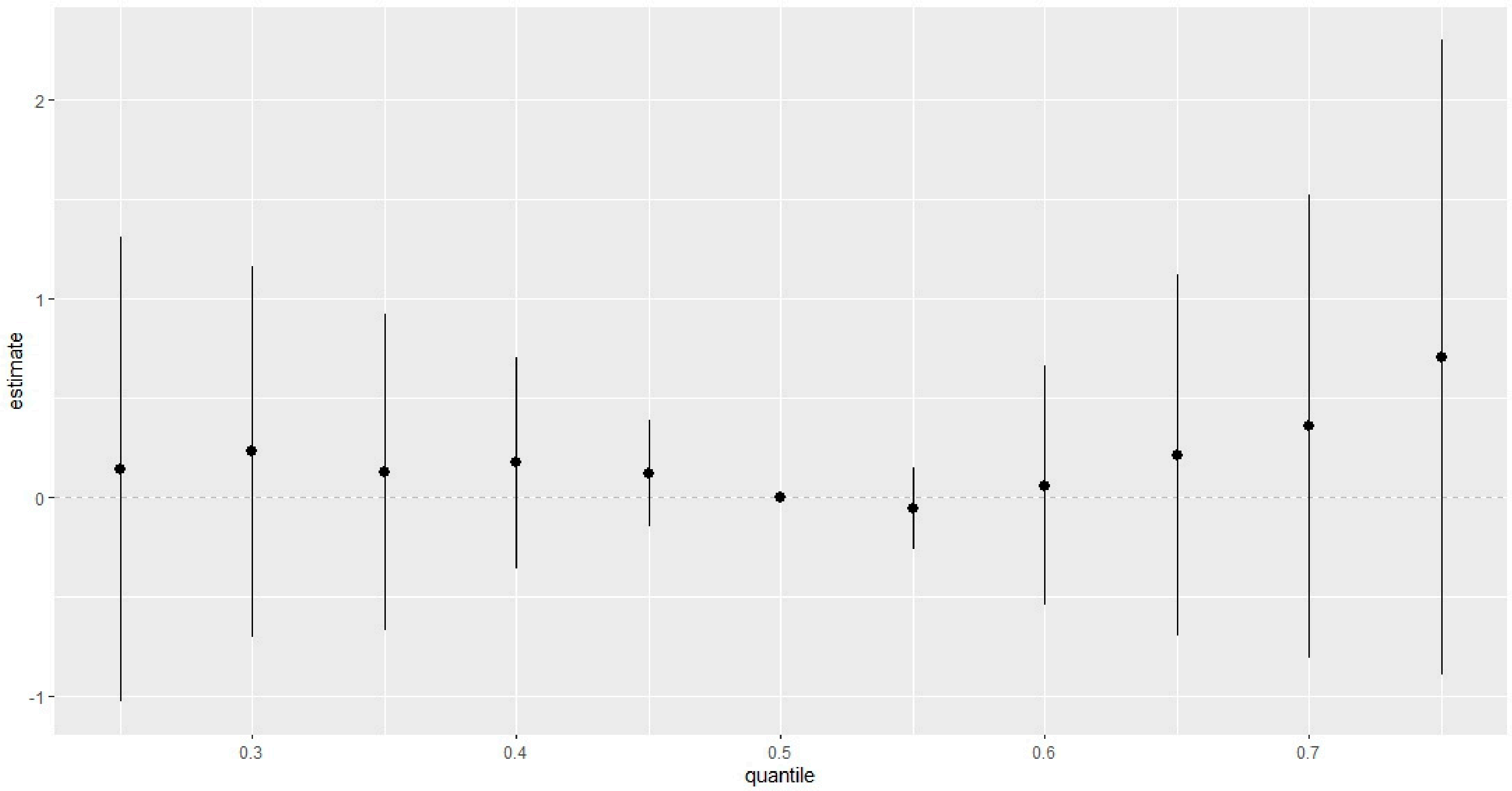

Note. Model was adjusted for the following covariates: second trimester urinary creatinine, maternal education, maternal marital status, maternal age, infant gestational age at birth, infant birthweight, and infant age at sample collection.

Figure S6. Overall effects (95% CIs) of the mixture of endocrine-disrupting chemicals on EAA in male infants (n = 118) using Bayesian kernel machine regression (BKMR) when all the exposures in the mixture at specific percentiles were compared to all the exposures at their 50th percentile.

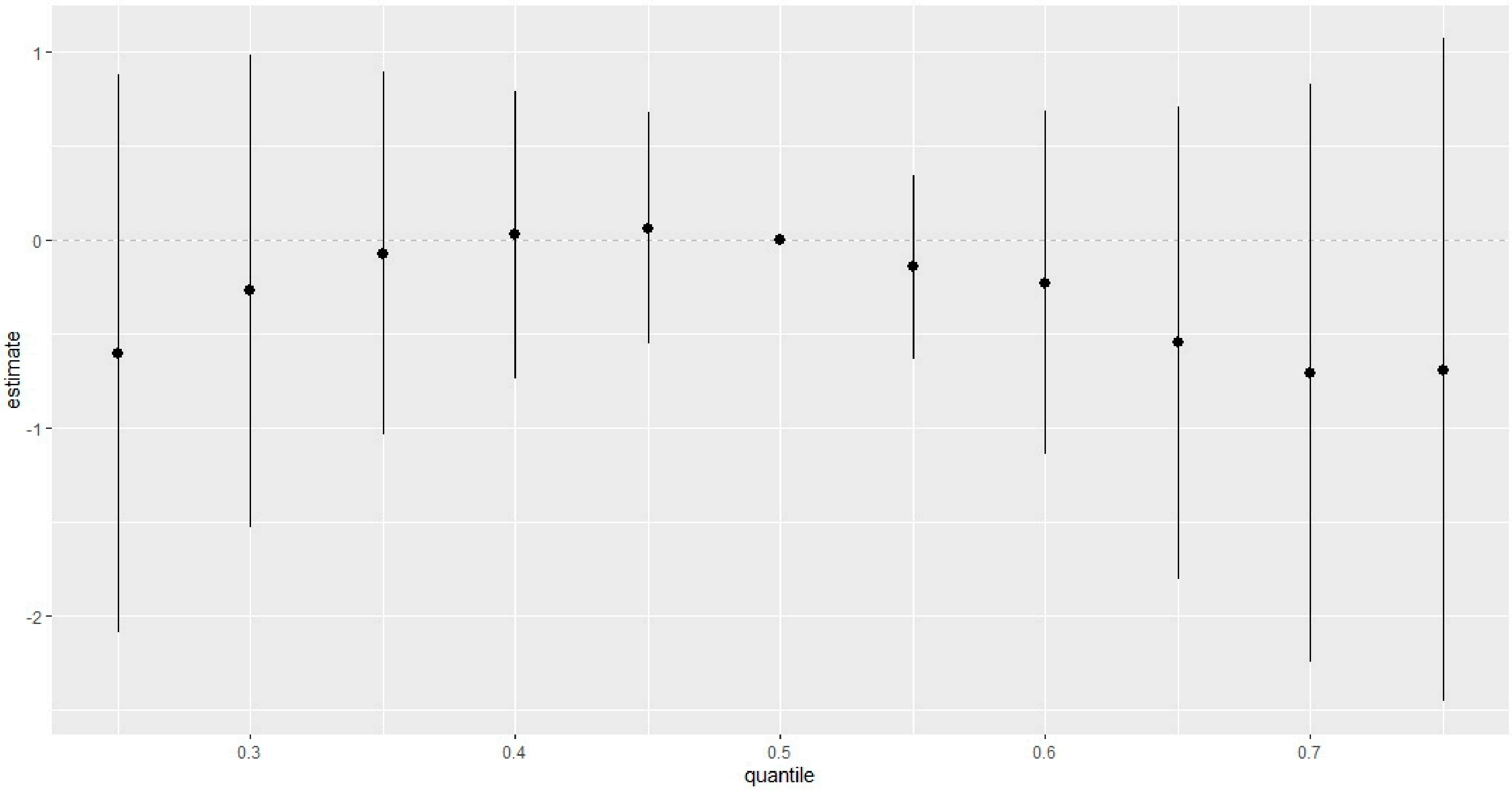

Note. Model was adjusted for the following covariates: second trimester urinary creatinine, maternal marital status, annual household income, maternal age, maternal pre-pregnancy body mass index (BMI), infant gestational age at birth, infant birthweight, and infant age at sample collection.

Figure S7. Participant flow diagram.

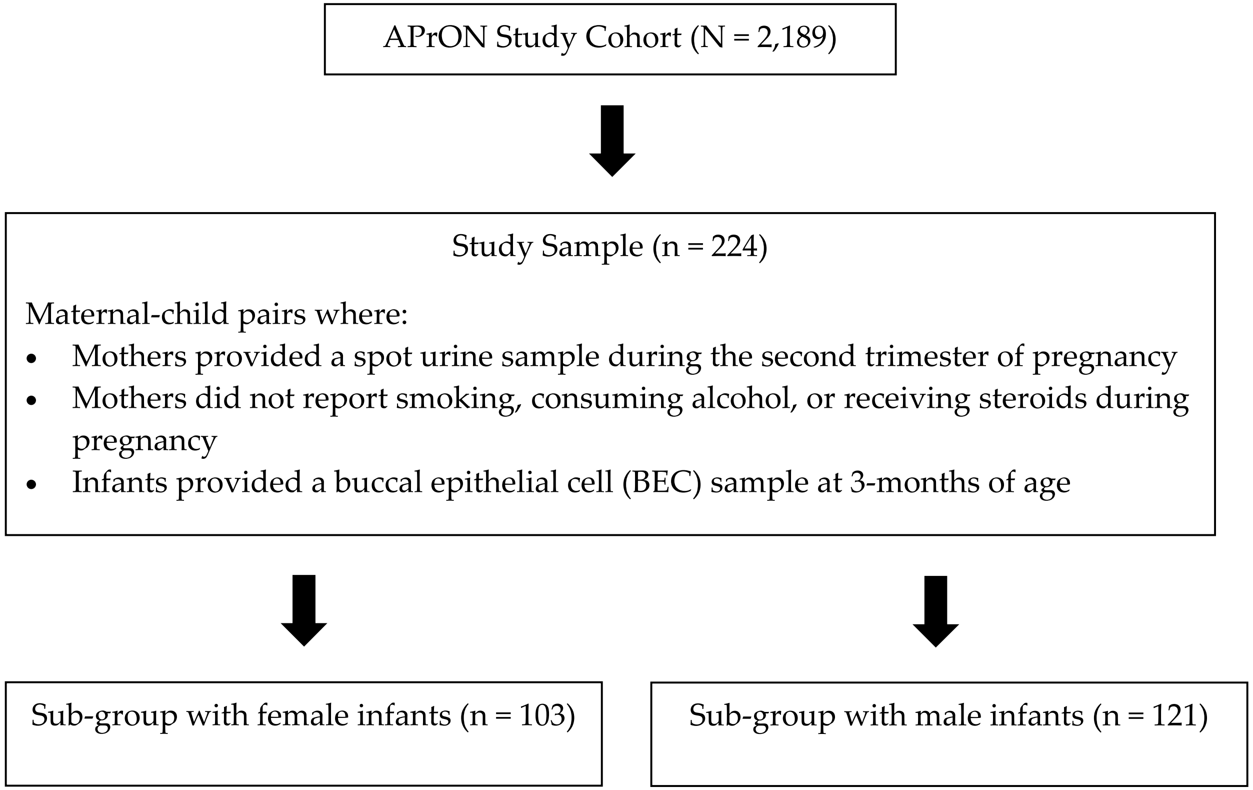

Figure S8. Directed acyclic graph (DAG) to depict the prior knowledge about pathways related to the research questions. The solid lines illustrate the links between variables included in the models. The dashed line indicates a possible indirect link (not tested).

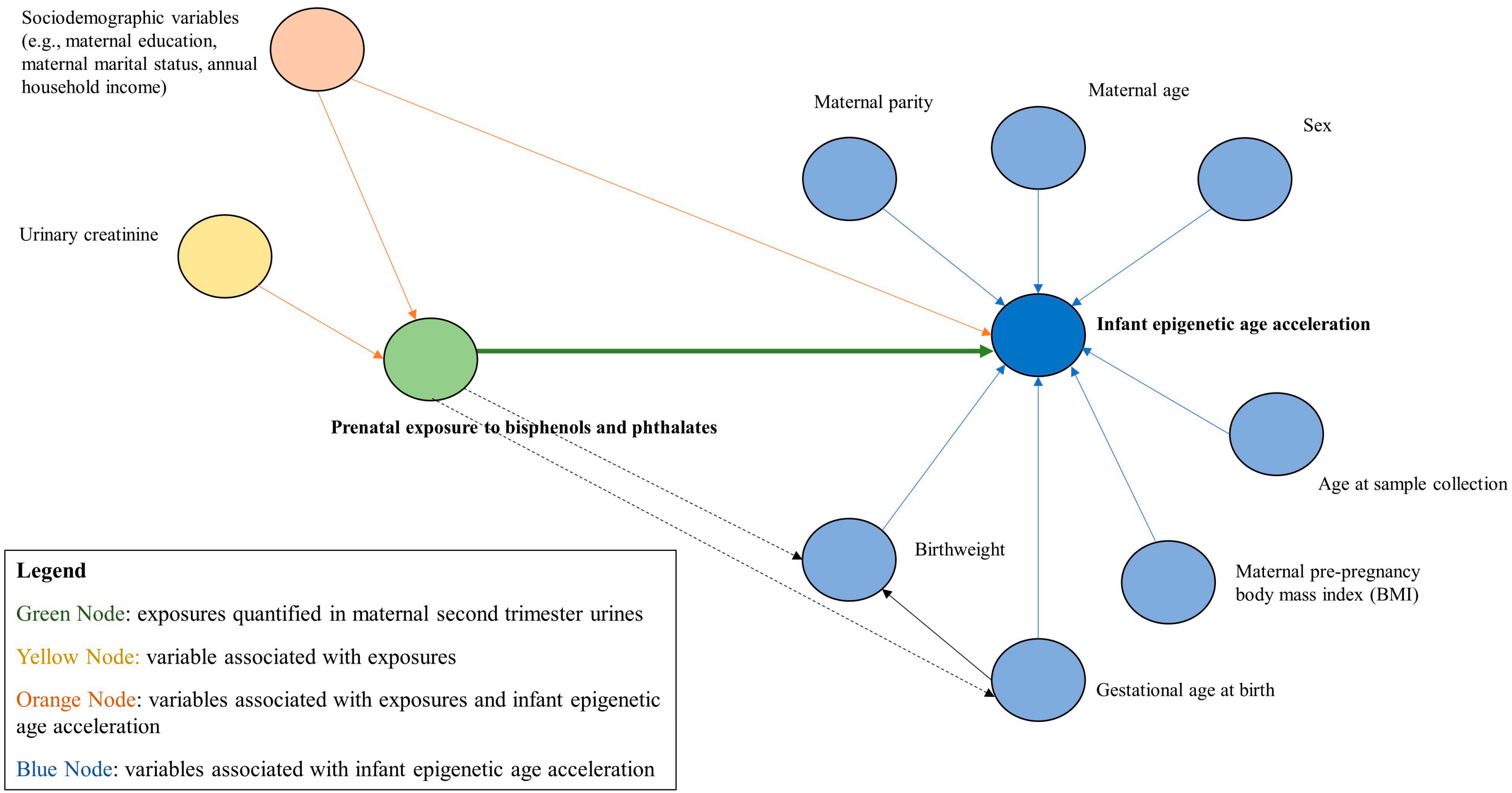

Table S1. Geometric means (GMs) of the bisphenol, phthalate molar sums, and phthalate metabolite concentrations in maternal second trimester urine samples for the sub-groups stratified by infant sex.

	Female infants (n = 103)	Male infants (n = 121)	*P*-value[a]
**Chemical**	**GM**	**GM**	
Bisphenols (μg/L)			
BPA[b]	1.59	1.21	0.11
BPS[c]	0.179	0.155	0.30
Phthalate Molar Sums (μmol/L)			
ΣLMWPs[d]	0.471	0.615	0.35
ΣHMWPs[i]	0.191	0.160	0.05*
ΣDEHP[j]	0.0909	0.0733	0.03*
Phthalate Metabolites (μg/L)			
MMP[e]	2.26	2.31	0.94
MEP[f]	48.4	68.3	0.40
MiBP[g]	9.43	9.69	0.40
MBP[h]	17.3	16.6	0.33

MEHP[k]	4.01	3.03	0.02*
MEHHP[l]	12.1	9.88	0.03*
MECPP[m]	16.6	14.0	0.06
MEOHP[n]	9.89	8.08	0.04*
MBzP[o]	7.21	7.61	0.92
MCOP[p]	13.1	11.2	0.87
MCNP[q]	1.04	0.923	0.29
MNP[r]	3.84	3.21	0.92

[a] *p*-values for F statistics from one-way analyses of variance (ANOVAs) for differences between sub-groups on continuous chemical concentrations (based on arithmetic means).
[b]BPA = bisphenol A
[c]BPS = bisphenol S
[d]ΣLMWPs = molar sum of low molecular weight phthalate metabolites; μmol/L; includes MMP, MEP, MiBP, and MBP
[e]MMP = mono-methyl phthalate
[f]MEP = mono-ethyl phthalate
[g]MiBP = mono-iso-butyl phthalate
[h]MBP = mono-n-butyl phthalate
[i]ΣHMWPs = molar sum of high molecular weight phthalate metabolites; μmol/L; includes MEHP, MEHHP, MEOHP, MECPP, MBzP, MCOP, MCNP, MNP
[j]ΣDEHP = molar sum of di(2-ethylhexyl) phthalate metabolites; μmol/L; includes MEHP, MEHHP, MEOHP, and MECPP
[k]MEHP = mono(2-ethylhexyl) phthalate

[l]MEHHP = mono(2-ethyl-5-hydroxyhexyl) phthalate
[m]MECPP = mono(2-ethyl-5-carboxypentyl) phthalate
[n]MEOHP = mono(2-ethyl-5-oxohexyl) phthalate
[o]MBzP = mono-benzyl phthalate
[p]MCOP = mono-carboxy-octyl phthalate
[q]MCNP = mono-carboxy-isononyl phthalate
[r]MNP = mono-isononyl phthalate

Table S2. Posterior inclusion probabilities (PIPs) for inclusion in the EAA model for female infants (n = 103) using Bayesian kernel machine regression (BKMR).

Chemical	Group[a]	Group PIP	Conditional PIP
BPA[b]	1	0.94	0.99
BPS[c]	1	0.94	0.01
MMP[d]	2	0.42	0.22
MEP[e]	2	0.42	0.45
MiBP[f]	2	0.42	0.20
MBP[g]	2	0.42	0.13
MEHP[h]	3	0.45	0.15
MEHHP[i]	3	0.45	0.12
MECPP[j]	3	0.45	0.21
MEOHP[k]	3	0.45	0.13

MBzP[l]	3	0.45	0.11
MCOP[m]	3	0.45	0.12
MCNP[n]	3	0.45	0.06
MNP[o]	3	0.45	0.12

[a]groups included in the hierarchical BKMR; the first group included 2 bisphenols, the second group included 4 low molecular weight phthalate metabolites, and third group included the 8 high molecular weight phthalate metabolites
[b]BPA = bisphenol A
[c]BPS = bisphenol S
[d]MMP = mono-methyl phthalate
[e]MEP = mono-ethyl phthalate
[f]MiBP = mono-iso-butyl phthalate
[g]MBP = mono-n-butyl phthalate
[h]MEHHP = mono(2-ethyl-5-hydroxyhexyl) phthalate
[i]MECPP = mono(2-ethyl-5-carboxypentyl) phthalate
[j]MEOHP = mono(2-ethyl-5-oxohexyl) phthalate
[k]MBzP = mono-benzyl phthalate
[l]MCOP = mono-carboxy-octyl phthalate
[m]MCNP = mono-carboxy-isononyl phthalate
[n]MEHP = mono(2-ethylhexyl) phthalate
[o]MNP = mono-isononyl phthalate
Note. Model was adjusted for the following covariates: second trimester urinary creatinine, maternal education, maternal marital status, maternal age, infant gestational age at birth, infant birthweight, and infant age at sample collection.

Table S3. Posterior inclusion probabilities (PIPs) for inclusion in the EAA model for male infants (n = 118) using Bayesian kernel machine regression (BKMR).

Chemical	Group[a]	Group PIP	Conditional PIP
BPA[b]	1	0.37	0.58
BPS[c]	1	0.37	0.42
MMP[d]	2	0.27	0.22
MEP[e]	2	0.27	0.23
MiBP[f]	2	0.27	0.37
MBP[g]	2	0.27	0.19
MEHP[h]	3	0.31	0.10
MEHHP[i]	3	0.31	0.14
MECPP[j]	3	0.31	0.13
MEOHP[k]	3	0.31	0.12

MBzP[l]	3	0.31	0.12
MCOP[m]	3	0.31	0.13
MCNP[n]	3	0.31	0.12
MNP[o]	3	0.31	0.13

[a]groups included in the hierarchical BKMR; the first group included 2 bisphenols, the second group included 4 low molecular weight phthalate metabolites, and third group included the 8 high molecular weight phthalate metabolites
[b]BPA = bisphenol A
[c]BPS = bisphenol S
[d]MMP = mono-methyl phthalate
[e]MEP = mono-ethyl phthalate
[f]MiBP = mono-iso-butyl phthalate
[g]MBP = mono-n-butyl phthalate
[h]MEHHP = mono(2-ethyl-5-hydroxyhexyl) phthalate
[i]MECPP = mono(2-ethyl-5-carboxypentyl) phthalate
[j]MEOHP = mono(2-ethyl-5-oxohexyl) phthalate
[k]MBzP = mono-benzyl phthalate
[l]MCOP = mono-carboxy-octyl phthalate
[m]MCNP = mono-carboxy-isononyl phthalate
[n]MEHP = mono(2-ethylhexyl) phthalate
[o]MNP = mono-isononyl phthalate

Note. Model was adjusted for the following covariates: second trimester urinary creatinine, maternal marital status, annual household income, maternal age, maternal pre-pregnancy body mass index (BMI), infant gestational age at birth, infant birthweight, and infant age at sample collection.

Table S4. Robust regression models, adjusted for infant sex, examining the associations between log2-transformed prenatal bisphenol and phthalate exposures and infant epigenetic age acceleration (EAA) as estimated by the Pediatric-Buccal-Epigenetic (PedBE) clock in the entire sample (n = 224).

**Chemical**	**PedBE EAA**<br>***B* (95% CI)**
Bisphenols (μg/L)	
BPA[a]	0.67 (0.29, 1.03)**
BPS[b]	−0.02 (−0.40, 0.37)
Phthalate Molar Sums (μmol/L)	
ΣLMWPs[c]	−0.07 (−0.43, 0.28)
ΣHMWPs[h]	0.05 (−0.45, 0.56)
ΣDEHP[i]	−0.09 (−0.55, 0.37)
Phthalate Metabolites (μg/L)	
MMP[d]	−0.10 (−0.57, 0.36)
MEP[e]	−0.08 (−0.34, 0.19)
MiBP[f]	−0.02 (−0.50, 0.46)
MBP[g]	0.01 (−0.51, 0.53)

MEHP[j]	−0.06 (−0.43, 0.32)
MEHHP[k]	−0.09 (−0.54, 0.37)
MECPP[l]	−0.05 (−0.53, 0.43)
MEOHP[m]	−0.11 (−0.58, 0.37)
MBzP[n]	0.10 (−0.33, 0.52)
MCOP[o]	0.00 (−0.25, 0.35)
MCNP[p]	−0.08 (−0.41, 0.25)
MNP[q]	−0.01 (−0.28, 0.26)

** $q \leq 0.05$
** $q < 0.10$
[a]BPA = bisphenol A
[b]BPS = bisphenol S
[c]ΣLMWPs = molar sum of low molecular weight phthalate metabolites; μmol/L; includes MMP, MEP, MiBP, and MBP
[d]MMP = mono-methyl phthalate
[e]MEP = mono-ethyl phthalate
[f]MiBP = mono-iso-butyl phthalate
[g]MBP = mono-n-butyl phthalate
[h]ΣHMWPs = molar sum of high molecular weight phthalate metabolites; μmol/L; includes MEHP, MEHHP, MEOHP, MECPP, MBzP, MCOP, MCNP, and MNP
[i]ΣDEHP = molar sum of di(2-ethylhexyl) phthalate metabolites; μmol/L; includes MEHP, MEHHP, MEOHP, and MECPP
[j]MEHP = mono(2-ethylhexyl) phthalate
[k]MEHHP = mono(2-ethyl-5-hydroxyhexyl) phthalate
[l]MECPP = mono(2-ethyl-5-carboxypentyl) phthalate
[m]MEOHP = mono(2-ethyl-5-oxohexyl) phthalate

[n]MBzP = mono-benzyl phthalate
[o]MCOP = mono-carboxy-octyl phthalate
[p]MCNP = mono-carboxy-isononyl phthalate
[q]MNP = mono-isononyl phthalate

Table S5. Sex-stratified robust regression models examining the associations between log2-transformed prenatal bisphenol and phthalate exposures and infant epigenetic age acceleration (EAA) as estimated by the Horvath pan-tissue epigenetic clock.

	**Female infants (n = 103)**	**Male infants (n = 121)**
**Chemical**	**EAA**<br>***B* (95% CI)**	**EAA**<br>***B* (95% CI)**
Bisphenols (μg/L)		
BPA[a]	0.003 (−0.05, 0.05)	−0.12 (−0.05, 0.06)
BPS[b]	0.02 (−0.03, 0.07)	0.02 (−0.03, 0.07)
Phthalate Molar Sums (μmol/L)		
ΣLMWPs[c]	−0.04 (−0.09, 0.02)	0.03 (−0.01, 0.08)
ΣHMWPs[h]	−0.03 (−0.09, 0.04)	0.01 (−0.06, 0.08)
ΣDEHP[i]	−0.04 (−0.09, 0.02)	0.01 (−0.06, 0.07)
Phthalate Metabolites (μg/L)		
MMP[d]	−0.02 (−0.08, 0.04)	−0.002 (−0.07, 0.06)
MEP[e]	−0.02 (−0.05, 0.02)	0.02 (−0.01, 0.05)
MiBP[f]	−0.02 (−0.10, 0.05)	0.01 (−0.05, 0.07)

MBP[g]	−0.05 (−0.12, 0.02)	0.05 (−0.02, 0.11)
MEHP[j]	−0.04 (−0.08, 0.01)	0.01 (−0.05, 0.06)
MEHHP[k]	−0.03 (−0.09, 0.02)	0.01 (−0.06, 0.08)
MECPP[l]	−0.03 (−0.09, 0.03)	−0.004 (−0.07, 0.06)
MEOHP[m]	−0.03 (−0.09, 0.03)	0.01 (−0.06, 0.07)
MBzP[n]	0.004 (−0.05, 0.06)	0.02 (−0.04, 0.08)
MBzP[o]	−0.04 (−0.09, 0.01)	−0.01 (−0.05, 0.04)
MCOP[p]	−0.02 (−0.07, 0.02)	−0.02 (−0.06, 0.02)
MCNP[q]	−0.04 (−0.07, −0.01)**	−0.01 (−0.04, 0.03)

** $q > 0.05$
[a]BPA = bisphenol A
[b]BPS = bisphenol S
[c]ΣLMWPs = molar sum of low molecular weight phthalate metabolites; μmol/L; includes MMP, MEP, MiBP, and MBP
[d]MMP = mono-methyl phthalate
[e]MEP = mono-ethyl phthalate
[f]MiBP = mono-iso-butyl phthalate
[g]MBP = mono-n-butyl phthalate
[h]ΣHMWPs = molar sum of high molecular weight phthalate metabolites; μmol/L; includes MEHP, MEHHP, MEOHP, MECPP, MBzP, MCOP, MCNP, and MNP
[i]ΣDEHP = molar sum of di(2-ethylhexyl) phthalate metabolites; μmol/L; includes MEHP, MEHHP, MEOHP, and MECPP
[j]MEHP = mono(2-ethylhexyl) phthalate

[k]MEHHP = mono(2-ethyl-5-hydroxyhexyl) phthalate
[l]MECPP = mono(2-ethyl-5-carboxypentyl) phthalate
[m]MEOHP = mono(2-ethyl-5-oxohexyl) phthalate
[n]MBzP = mono-benzyl phthalate
[o]MCOP = mono-carboxy-octyl phthalate
[p]MCNP = mono-carboxy-isononyl phthalate
[q]MNP = mono-isononyl phthalate
